# Surgeon’s preference of subcutaneous tissue resection: most important factor for short-term complications in subcutaneous implant placement after mastectomy—results of a cohort study

**DOI:** 10.1007/s00404-020-05481-x

**Published:** 2020-03-09

**Authors:** André Pfob, Vivian Koelbel, Florian Schuetz, Manuel Feißt, Maria Blumenstein, André Hennigs, Michael Golatta, Joerg Heil

**Affiliations:** 1 Department of Gynecology, Breast Center, Heidelberg University, Im Neuenheimer Feld 440, 69120 Heidelberg, Germany; 2 Institute of Medical Biometry and Informatics, Heidelberg University, Heidelberg, Germany

**Keywords:** Subcutaneous breast implant, Mastectomy, Shared decision making, Drain management

## Abstract

**Purpose:**

Little is known about the reason of high short-term complication rates after the subcutaneous placement of breast implants or expanders after mastectomy without biological matrices or synthetic meshes. This study aims to evaluate complications and their risk factors to develop guidelines for decreasing complication rates.

**Methods:**

We included all cases of mastectomy followed by subcutaneous implant or expander placement between 06/2017 and 05/2018 (*n* = 92). Mean follow-up time was 12 months.

**Results:**

Explantation occurred in 15 cases (16.3%). The surgeon’s preference for moderate vs. radical subcutaneous tissue resection had a significant influence on explantation rates (*p* = 0.026), impaired wound healing or infection (requiring surgery) (*p* = 0.029, *p* = 0.003 respectively) and major complications (*p* = 0.018).

Multivariate analysis revealed significant influence on complication rates for radical subcutaneous tissue resection (*p* up to 0.003), higher implant volume (*p* up to 0.023), higher drain volume during the last 24 h (*p* = 0.049), higher resection weight (*p* = 0.035) and incision type (*p* = 0.011).

**Conclusion:**

Based on the significant risk factors we suggest the following guidelines to decrease complication rates: favoring thicker skin envelopes after surgical preparation, using smaller implants, removing drains based on a low output volume during the last 24 h and no use of periareolar incision with extension medial or lateral. We should consider ADMs for subcutaneous one-stage reconstructions.

The individual surgeon’s preference of subcutaneous tissue resection is of highest relevance for short-term complications—this has to be part of internal team discussions and should be considered in future trials for comparable results.

## Introduction

During past decades subcutaneous implant placement has been considered to be inferior to a subpectoral approach due to high complication rates (especially capsular fibrosis, infection, and skin necrosis) [1, 2]. However, the subcutaneous placement seems to be superior in terms of functional and aesthetic outcome [3]. The introduction of new surgery techniques (skin- and nipple-sparing mastectomy), new indications and additional devices like meshes require a reevaluation of complication rates in subcutaneous implant or expander placement.

Recently, several small studies investigated the complication rates in subcutaneous placement. Data are not conclusive as complication rates largely vary (explantation ranges from 0 to 18%, seroma 2–23%, nipple or skin necrosis 1–28%) [4–18]. There are three main reasons for this big variance:

- First, inconsistent selection criteria and the inconsistent use of acellular dermal matrices or meshes among these studies, which leads to heterogeneous patient groups being compared.
- Second, potential factors like the surgeon, drain management, prophylactic antibiosis and neoadjuvant chemotherapy (NACT) are barely considered, and there are little data on the influence of smoking, previous radiation of the breast area and axillary lymph node dissection (ALND), too.
- Third, limited data are available for approaches without acellular dermal matrices (ADM) and synthetical meshes in subcutaneous breast implant placement.

Therefore, besides providing further data on complication rates, this study aims to determine risk factors of complications in subcutaneous implant or expander placement without additional heterologous materials in breast surgery. Knowing these risk factors would allow developing guidelines for minimizing complications, and informing patients and surgeons better.

## Patients and methods

This explorative, single-center, retrospective cohort study includes all female patients from June 2017 to May 2018 undergoing therapeutic or prophylactic mastectomy followed by the placement of a subcutaneous implant or expander; either to finally reconstruct the breast or to prepare delayed-immediate autologous reconstruction. Ninty-two cases were identified. The mean follow-up time was 12 months.

### In- and exclusion criteria

Inclusion criteria were therapeutic or prophylactic mastectomy followed by the placement of a subcutaneous implant or expander; either to finally reconstruct the breast or to prepare delayed-immediate autologous reconstruction.

Exclusion criteria were definied as previous breast reconstruction or revision.

### Surgery technique

Surgeries were performed by six experienced oncoplastic breast surgeons. Please note that no meshes or ADMs were used.

Drains were supposed to be removed when the output volume during the last 24 h is < 30 ml.

Single-shot prophylactic antibiosis was routinely used—based on the physician’s evaluation antibiosis could be extended for several days (see Table 1).

**Table 1:** Study cohort characteristics

Variable	Mean (standard deviation)	Median (quartile 1; quartile 3)	Total (*n*)
Age (years)	46.0 (11.8)	46.5 (37.5; 52.5)	92
BMI (kg/m^2^)	25.8 (6.0)	23.7 (21.9; 28.4)	91
Mastectomy weight (g)	416.4 (240.1)	385.0 (260.0; 496.0)	89
Implant volume (ml)	397.2 (160.2)	375.0 (275.0; 470.0)	85
Expander volume intraoperatively (ml)	143.3 (48.0)	150.0 (150.0; 180.0)	6
Drain duration (days)	5.9 (3.1)	5.0 (4.0; 8.0)	85
Drain cumulative volume (ml)	458.3 (521.9)	285.0 (140.0; 585.0)	85
Drain volume last 24 h (ml)	16.7 (11.5)	20.0 (6.5; 21.5)	84
Prophylactic antibiotics duration (days)	2.8 (3.3)	1.0 (1.0; 4.0)	92

Variable	Yes (%)	No (%)	Total (*n*)
Smoking	9 (9.8)	83 (90.2)	92
Diabetes	2 (2.2)	90 (97.8)	92
Breast cancer	62 (67.4)	30 (32.6)	92
Previous radiation of the Breast area	10 (10.9)	82 (89.1)	92
NACT	37 (40.2)	55 (59.8)	92
ALND	18 (19.6)	74 (80.4)	92
SLNB	39 (42.4)	53 (57.6)	92
Incision type			90
Fishmouth	55 (61.1)		
Submammary	11 (12.2)		
Hemi-periareolar	12 (13.3)		
Hemi-periareolar with extension medial or lateral	11 (12.2)		
Other	1 (0.8)		
Skin-sparing mastectomy	56	34	90
Nipple-sparing mastectomy	34	56	90
Local recurrence after 12 months follow-up	2 (3.2)	60 (96.8)	62

*ALND* axillary lymph node dissection, *SLN*B sentinel lymphe node biopsy, *NACT* neoadjuvant chemotherapy

#### Nipple-sparing mastectomy (NSM)

Eligibility criteria for NSM were: mastectomy indication, tumor size (< 3.5 cm), tumor location (> 2 cm distance from nipple-areola complex), no previous NACT, no Paget disease, no inflammatory breast cancer. NSM was performed using a submammary or hemi-periareolar (with extension medial or lateral) incision. The tissue beneath the nipple was pathologically evaluated intraoperatively by frozen sections to rule out any tumor involvement of the nipple. The surgeon clinically evaluated skin flap viability and thickness during surgery (visual inspection and capillary refill). For surgical margins, see the discussion section.

#### Skin-sparing mastectomy (SSM)

Eligibility criteria for SSM were: mastectomy indication, tumor stage I–II (possible for stage III), and no inflammatory breast cancer. SSM was performed using periareolar (fishmouth) incision. The surgeon clinically evaluated skin flap viability and thickness during surgery (visual inspection and capillary refill). For surgical margins, see the discussion section.

### Statistical analysis

First, we analyzed influences by univariate statistics. Subsequently, significant results were considered in a multivariate logistic regression model (forward-stepwise, *α* = 0.25) for each dependent variable (= complication). Metric data were centralized and standardized for comparable effect sizes; missing data were excluded from analysis.

Aspirated seromas were considered as a nominal variable, and nipple necrosis was only considered if more than 50% of the nipple was affected. The occurrence of a major complication was defined as complications requiring surgery.

To evaluate the question of whether surgery can prevent the loss of an implant once a major complication has occurred, we identified saved implants by major complications undergoing surgery but without implant loss. Not saved implants are defined as those that needed to be explanted despite (multiple) surgeries after a major complication.

Because of the explorative study design adjustment for multiplicity was not done. Thus, *p* values < 0.05 are considered to be statistically significant in a descriptive sense. The effect size of the risk factors is illustrated by odds ratio (OR).

Due to low caseload results of table four are shown descriptively, we added p values of the chi-square test for illustration.

SPSS software, version 25.0 (SPSS, Chicago, IL) was used for all statistical analysis.

## Ethical approval

The Heidelberg ethical committee approved this study (S-585/2018).

The study was deemed to be potentially valuable and without risk for patients, including anonymized, retrospective analysis of routinely collected data. Consequently and in compliance with § 13 Abs. 1 LDSG BW, the ethics committee of the University of Heidelberg did not request approval of informed consent for this designated analysis.

## Results

### Descriptive distribution of study cohort

The distribution of risk factors among the 92 cases is shown in Table 1. The average age was 46.0 years (standard deviation SD 11.8); 67.4% of the implant or expander placements were performed because of breast cancer, the other 32.6% due to prophylactic mastectomy—the high rate of prophylactic mastectomies might explain the rather young average of age. Wound drains remained for a mean of 5.9 days (SD 3.1), producing a mean cumulative volume of 458.3 ml (SD 521.9); the mean volume during the last 24 h was 16.7 ml (SD 11.5)—drain management seemed to be highly variable, illustrated by the high standard deviations. Only two patients were diagnosed with diabetes, and only six reconstructions used expanders.

Complication rates are shown in Table 2. Explantation of the implant occurred in 15 cases (16.3%).

**Table 2:** Complication rates in subcutaneous implant placement (*n* = 92)

Complication	Yes (%)	No (%)
Explantation	15 (16.3)	77 (83.7)
One-stage reconstruction	14 (16.3)	72 (83.7)
Two-stage reconstruction	1 (16.7)	5 (83.3)
Seroma aspirated (nominal)	17 (18.5)	75 (81.5)
Hematoma or bleeding requiring surgery	5 (5.4)	87 (94.6)
Impaired wound healing or infection	23 (25.0)	69 (75.0)
Impaired wound healing or infection requiring surgery	19 (20.7)	73 (79.3)
Major nipplenecrosis (> 50%)	5 (5.4)	87 (94.6)
Major complication (requiring surgery)	26 (28.3)	66 (71.7)
Minor complication (not requiring surgery)	31 (33.7)	61 (66.3)

Table 3 shows a summary of the risk factors associated with complications.

**Table 3:** Risk factors associated with complications in multivariate analysis for subcutaneous implant placement

Dependent variable	Independent variable	*p* value	OR (95% confidence interval)
Explantation	Implant volume	0.023	2.022 (1.101–3.712)
	Surgeon	0.026	0.087 (0.010–0.743)
Seroma aspirated (nominal)	Drain volume last 24 h before removal	0.049	1.724 (1.002–2.964)
Impaired wound healing or infection requiring surgery	Surgeon	0.003	0.037 (0.004–0.326)
	Resection weight	0.035	2.057 (1.051–4.027)
Nipple necrosis	Incision type (periareolar with extension medial or lateral)	0.011	22.568 (2.030–250.864)

*OR* odds ratio

Multivariate analysis revealed a significant influence of the surgeon, higher drain volume during the last 24 h, higher implant volumes, higher resection weight, and incision type:

The surgeon represents the most common risk factor and also the one with the largest effect sizes. The surgeon had a significant influence on explantation rates (*p* = 0.026, OR 0.087), impaired wound healing or infection (requiring surgery) (*p* = 0.029, OR 0.268; *p* = 0.003, OR 0.037 respectively) and major complication (*p* = 0.018, OR 0.261).

Higher implant volumes were another common risk factor and significantly associated with explantation (*p* = 0.023, OR 2.022), impaired wound healing or infection (*p* = 0.007, OR 2.177) and minor complication (*p* = 0.012, OR 1.872).

A periareolar incision without extension did not significantly affect nipple necrosis—but a periareolar incision with extension medial or lateral did (*p* = 0.011, OR 22.568).

Complication rates were significantly higher (in univariate, not in multivariate analysis) for high drain volume during the last 24 h (*p* = 0.045 for explantation) and when the implant was used as a temporary spacer in terms of delayed-immediate autologous reconstruction: infection or impaired healing (*p* = 0.026, OR 3.153), infection or impaired healing requiring surgery (*p* = 0.020, OR 3.794), major complication (*p* = 0.017, OR 3.250), minor complication (*p* = 0.035, OR 2.618) and explantation (*p* = 0.047, OR 3.478).

There was no influence for skin- vs. nipple-sparing mastectomy on complication rates.

Some complications on their own were associated with loss of implant: Impaired wound healing or infection (requiring surgery), major complications and even seroma significantly correlated with explantation, whereas hematoma or bleeding requiring surgery were never associated with explantation (see Table 4). Impaired wound healing or infection requiring surgery resulted in implant loss in 78.9% of these cases.

**Table 4:** Complications associated with explantation in subcutaneous implant placement

Complication	Explantation		*p* value
	No (% as rows)	Yes (% as rows)	
Seroma aspirated (nominal)			
No	66 (88.0)	9 (12.0)	0.030
Yes	11 (64.7)	6 (35.3)	
Hematoma or bleeding requiring surgery			
No	72 (82.8)	15 (17.2)	0.587
Yes	5 (100.0)	0 (0.0)	
Impaired wound healing or infection			
No	69 (100.0)	0 (0.0)	< 0.001
Yes	8 (34.8)	15 (65.2)	
Impaired wound healing or infection requiring surgery			
No	77 (93.9)	5 (6.1)	< 0.001
Yes	4 (21.1)	15 (78.9)	
Nipplenecrosis			
No	74 (85.1)	13 (14.9)	0.185
Yes	3 (60.0)	2 (40.0)	
Major complication			
No	66 (100.0)	0 (0.0	< 0.001
Yes	11 (42.3)	15 (57.7)	

Another controversial question is whether surgery can prevent the loss of an implant once a major complication has occurred. Very relevant risk factors in losing the implant after a surgical intervention because of a major complication were previous radiation of the breast (implant loss in 100%), NACT (92.3%), mastectomy due to breast cancer (80.0%) and impaired wound healing or infection (78.9%). However, after nipple necrosis the implant could be preserved by surgery with a chance of 60%.

After 12 months follow-up, 2 patients (3.2%) showed local recurrence (43 of the 62 breast cancer patients underwent follow-up in our hospital, the other 19 in an outpatient setting).

## Discussion

We found several risk factors for complications in subcutaneous implant or expander placement. We discuss their significance in the following to develop guidelines for subcutaneous implant placement.

### Significance of the surgeon

The surgeon was found to be the most common risk factor and also the one with the largest effect sizes. Six equally experienced breast surgeons with comparable caseload (about 20 per year) performed the breast reconstructions, but a team conference revealed different opinions regarding the radicality of subcutaneous tissue resection:

Some surgeons favored a more radical approach, removing more tissue beneath the skin to maximize oncologic safety but thus leaving thinner skin envelopes. Others preferred to leave more fat tissue beneath the skin, which results in thicker skin envelopes but might increase the risk of leaving gland tissue behind.

**Fig. 1:**
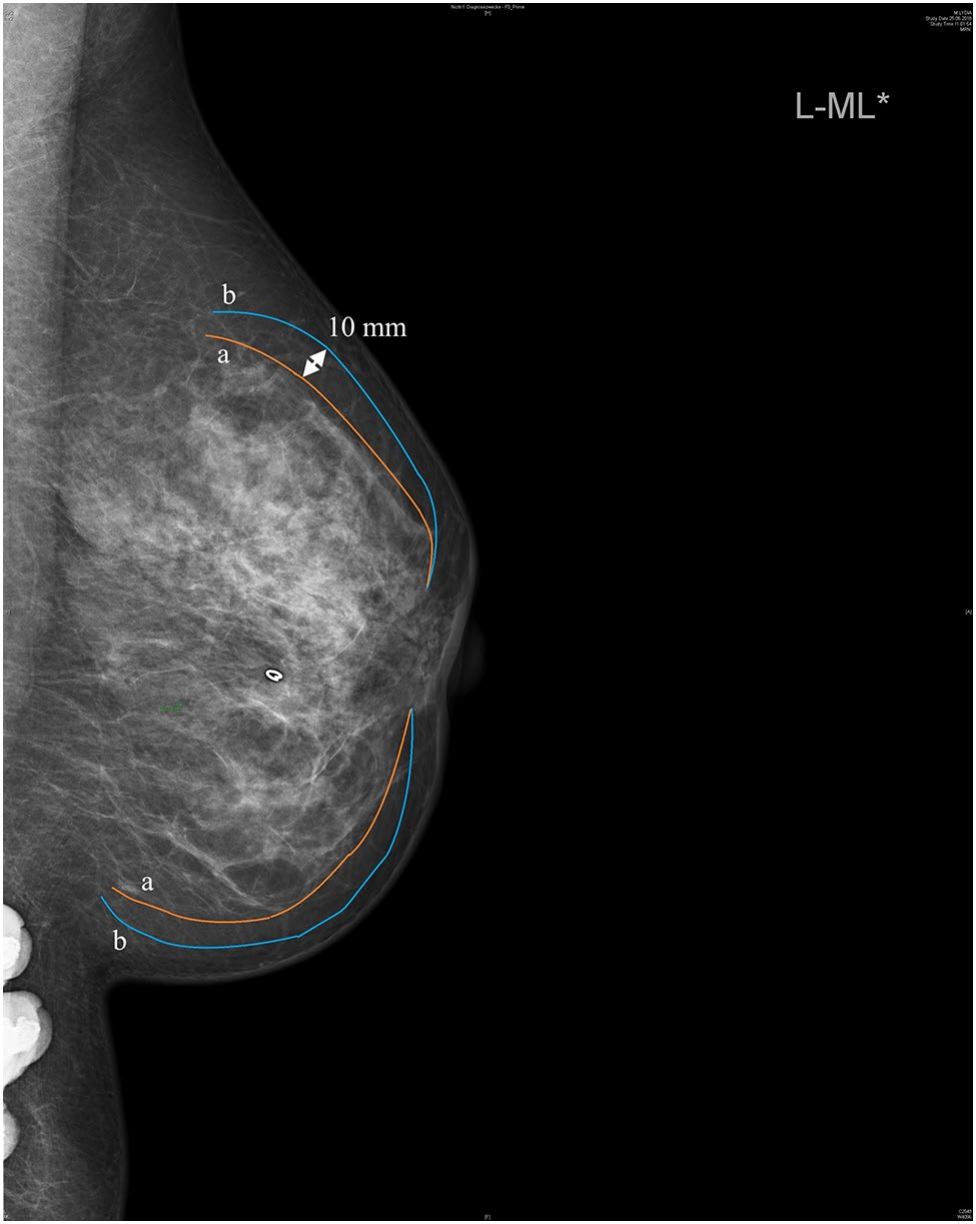
Resection margins for moderate vs. radical subcutaneous tissue resection. The six breast surgeons were asked to illustrate their typical resection margins for the same patient`s preoperative mammography (central tumour marked with a clip). Two approaches could be separated: a radical one (**b**, average resection margins of 4 surgeons) and a moderate one (**a**, average resection margins of 2 surgeons). Arrow illustrates the maximum distance between the two groups

We asked the surgeons to illustrate their typical resection margins for the same patient’s mammography (Fig. 1). Based on their average resection margins, we divided the six surgeons into two groups: a radical and a moderate group. Resection margins match near the anterior nipple area and the posterior end of the gland area, but in between the skin flap thickness differs up to 10 mm.

**Fig. 2:**
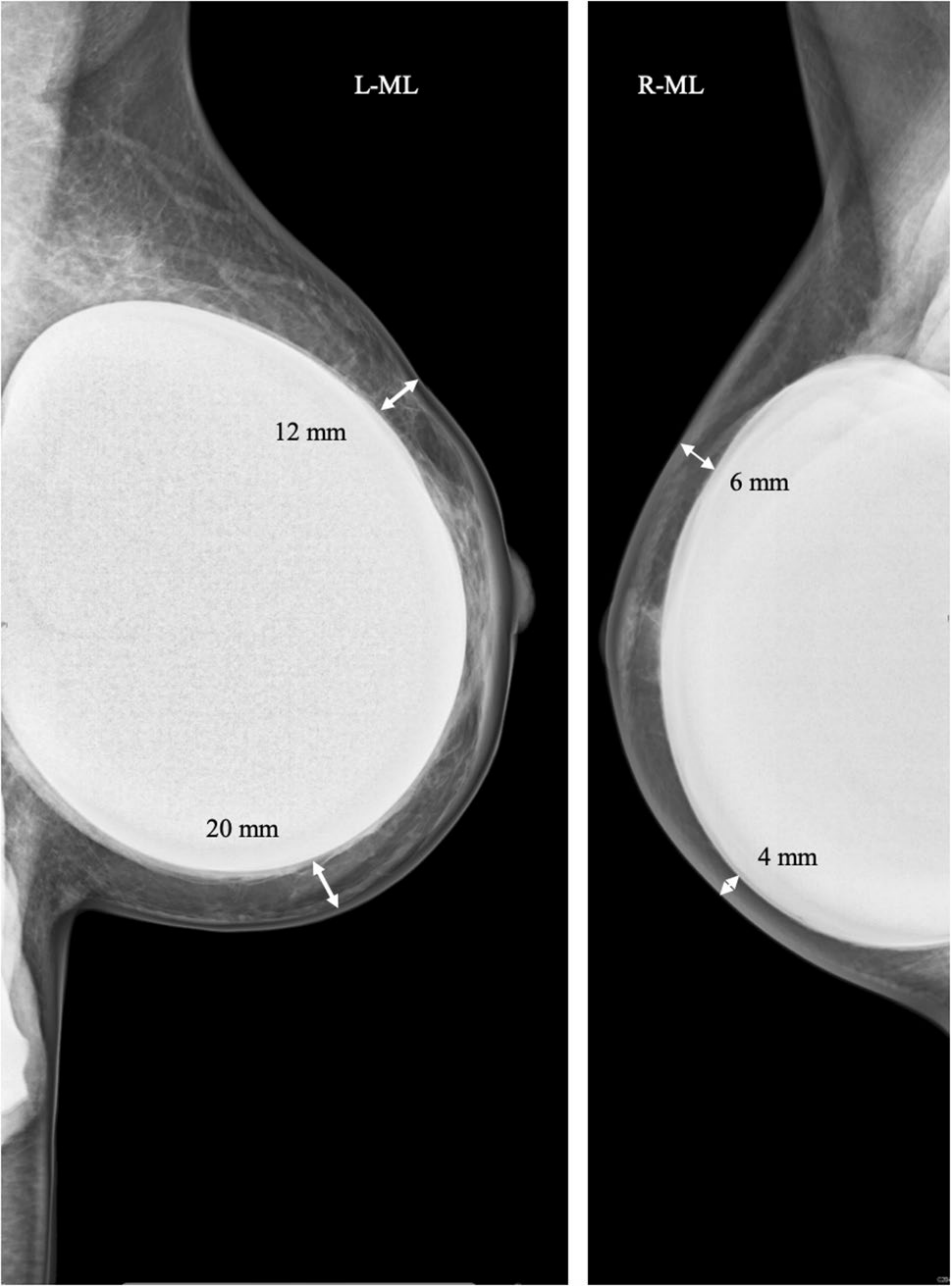
Postoperative skin flap thickness for moderate vs. radical subcutaneous tissue resection. Exemplary postoperative skin flap thickness after moderate subcutaneous resection (left, 11 months post-surgery) and radical subcutaneous resection (right, 4 months post-surgery); arrows illustrate skin flap thickness

Figure 2 exemplarily compares the actual postoperative thickness of the skin envelope between the radical and the moderate group: Results are similar to the above discussed planned resection margins—the radical approach leads to thinner skin flaps between the anterior nipple area and the posterior end of the gland tissue.

Our data shows that the radical group (favoring to remove more subcutaneous tissue) had significantly more complications (explantations *p* = 0.026, impaired wound healing or infection (requiring surgery) *p* = 0.007, *p* = 0.003 respectively). Harming the superficial blood supply in the subcutaneous and subdermal layers—and thus impairing skinflap quality [19–24]—is a likely reason. Skinflap quality has been identified as the leading factor for ischemic complications for immediate breast reconstructions (*n* = 86 of 92 in our study population) [25–27]. Also the interesting finding that a periareolar incision without extension did not significantly affect nipple necrosis but a periareolar incision with extension medial or lateral did, indicates that even a more extensive incision might harm that superficial blood supply and thus leads to more complications. To protect the delecate blood supply during surgery, the benefit of detecting the skin blood supply (e.g. by indocyanine green fluorescence angiography) might be assessed in future trials.

However, even if a radical subcutaneous tissue resection leads to more complications: removing less tissue has always been associated with major oncologic concerns because it increases the risk of leaving gland tissue behind. In our study population, there were two cases (3.2%) of local recurrence after 12 months follow-up: one case in the radical surgeon group and one case in the moderate surgeon group. For our data, the moderate resection of subcutaneous tissue does not decrease the oncologic safety while decreasing complications after subcutaneous implant placement. But leaving more subcutaneous tissue may lead to a higher recurrence rate. Prospective and at best randomized trials are needed to confirm this hypothesis.

To our knowledge, this study shows for the first time that radical subcutaneous tissue resection results not only in ischemic complications but promotes other major complications, too. Our study also discusses for the first time that flap thickness is not only a predetermined patient-specific factor but actively and willingly influenced by the surgeon’s mastectomy technique, too. This has to be part of internal team discussions and should be considered in future trials for comparable results.

### Significance of wound drain management

The results of our study suggest that complications like explantation and seroma could be decreased by adapted wound drain management. Several studies dealt with drain management after mastectomy but show inconsistent results regarding cumulative drain volume and the day of removal after surgery [28–32]. Our data suggest that drains should remain until a defined cut off volume (to be defined in prospective trials) during the last 24 h is reached, rather than adhering to a fix timeframe or cumulative volume after surgery.

High drain volume during the last 24 h was significantly associated with explantation and seroma; besides the longer the drain remains, the higher is the drain volume during the last 24 h (*p* = 0.001). This indicates that drains are often removed prematurely. One might conclude that surgeons must be more patient with these patients, and drains have to remain longer.

Several studies found a correlation between drain duration and infections. However, this influence could only be observed for a drain duration of 21 days or longer [33–35]. The drain duration of our study population was much shorter (Q1 5 days, Q3 8 days)—therefore, waiting a few more days before removing the drains is unlikely to cause significant more infections.

### Significance of implant volume

This study showed a significant impact of higher implant volume on explantation, impaired healing or wound infection and minor complications. Swanson et al. found no influence on implant volume on complications in a cohort of non-breast cancer patients [36]. The effect should be further investigated especially in breast cancer patients whose immunosystem is impaired compared to non-breast cancer patients. The effect might be mediated by a larger wound surface in bigger breasts promoting infections and impaired wound healing.

### Significance of antibiotics duration

Recent studies for subcutaneous placement observed that longer antibiotics duration is associated with lower complications (especially infections) [4–18]—we could not see this effect in our study for two reasons: First, intraoperative single shot antibiosis was predominantly used in our study (*n* = 61, 66.3%) and longer durations rarely occurred (maximum of 16 days in one case). Second, the effect size of antibiotics duration on complication rates might be too small compared to the other risk factors (especially the surgeon) which were only considered in our study.

### Significance of patient selection criteria

Current implant studies often exclude patients with risk factors anticipated with complications [4–18]. We could not observe any influence of selection criteria like NACT, smoking, BMI, previous radiation of the breast area (either due to previous breast cancer or other malignancies), or skin- vs. nipple-sparing mastectomy on higher complication rates. However, we saw that previous radiation of the breast area, NACT, breast cancer, and impaired wound healing or infection impair the chances of surgically saving the implant once a major complication occurred. If patients show one of these predictors after a major complication, it should be intensely evaluated with them whether it is worth trying a surgical attempt to save the implant.

### Significance of acellular dermal matrices

Regarding the ongoing discussion of whether or not to use acellular dermal matrices and meshes in a subcutaneous breast or expander placement, the findings of this study may help to inform this discussion. Salibian et al. 2017 (the only other known study population for a subcutaneous breast implant or expander placement without ADMs or meshes) found an explantation rate of 5.6% for a two-stage approach [13]. As our study mainly consists of one-stage reconstructions (*n* = 86 of 92) and showed an explantation rate of 16.3% (14 of 86), one might conclude that biological matrices and synthetic meshes should be used especially for subcutaneous one-stage reconstructions. The risk for harming the subcutaneous blood supply might be increased especially for one-stage (compared to two-stage) subcutaneous implant placement due to higher skin tension—acellular dermal matrices and meshes might help to preserve this blood supply by decreasing the tension. Further studies need to verify this hypothesis.

### Study limitations and bias

Shortly after our anaylsis there was a safety recall for some of the implants we used in our study due to breast implant-associated anaplastic large cell lymphoma. However, it is unlikely that this specific risk impairs the general observations of our study, especially because we observed only short-term complications.

The samples size limits our study but large effect sizes have already became obvious for our sample size. We addressed possible confirmation bias by performing and reporting the initial statistical analysis by two independent researchers.

### Guidelines for subcutaneous implant placement

The results of our study suggest the following guidelines to decrease complication rates in subcutaneous implant placement without ADMs or meshes: favoring thicker skin envelopes after surgical preparation (while keeping oncologic safety in mind), using smaller implants, removing drains based on a low output volume during the last 24 h and no use of periareolar incision with extension medial or lateral. For one-stage subcutaneous reconstructions, we should consider ADMs or meshes.

In future trials and trial comparisons, the identified factors (especially the surgeon’s preference for subcutaneous tissue resection) should be considered at first as they have a major impact on complication rates.

## Conclusion

The findings of this study help in clinical routine by informing patients and their physicians about what to expect from the subcutaneous placement of implants because prospective and at best randomized data are not available for a relevant number of questions. The individual surgeon’s preference of subcutaneous tissue resection is of highest relevance for short-term complications—this has to be part of internal team discussions and should be considered in future trials for comparable results, too. We suppose that especially drain management and the use of covering devices like ADMs and meshes should be investigated more rigorously to reduce complications after subcutaneous implant and expander placement.

## Data Availability

The datasets generated during and/or analysed during the current study are available in the Mendely repository, https://data.mendeley.com/datasets/vx4kx2bwyr/1.

## Abbreviations

ADM: Acellular dermal matrices
ALND: Axillary lymph node dissection
NACT: Neoadjuvant chemotherapy
NSM: Nipple-sparing mastectomy
OR: Odds ratio
SD: Standard deviation
SSM: Skin-sparing mastectomy
